# Associations of inflammatory cytokines with inflammatory bowel disease: a Mendelian randomization study

**DOI:** 10.3389/fimmu.2023.1327879

**Published:** 2024-01-15

**Authors:** Zhaoxiang Song, Xiangyu Li, Jinlin Xie, Fei Han, Nan Wang, Yuhan Hou, Jianning Yao

**Affiliations:** Department of Gastroenterology, The First Affiliated Hospital of Zhengzhou University, ZhengZhou, China

**Keywords:** inflammatory cytokines, biomarkers, inflammatory bowel disease, Mendelian randomization, GWAS

## Abstract

**Objectives:**

Previous studies have confirmed a link between specific inflammatory cytokines and inflammatory bowel disease (IBD), but the causal relationship between them is not completely clear. This Mendelian Randomization (MR) study aims to evaluate the causal relationship between 18 inflammatory cytokines and inflammatory bowel disease.

**Method:**

Two-sample Mendelian randomization utilized genetic variances associated with IBD from two extensive publicly available genome-wide association studies (GWAS) (Crohn’s Disease (CD): 12,194 cases and 28,072 controls; Ulcerative Colitis (UC): 12,336 cases and 33,609 controls). The data of inflammatory cytokines was acquired from a GWAS including 8,293 healthy participants. We used inverse variance weighted method, MR-Egger, weighted median, simple model and weighted model to evaluate the causal relationship between inflammatory cytokines and IBD. Sensitivity analysis includes heterogeneity and pleiotropy analysis to evaluate the robustness of the results.

**Results:**

The findings indicated suggestive positive associations between Interleukin-13 (IL-13) and macrophage migration inhibitory factor (MIF) with CD (odds ratio, OR: 1.101, 95%CI: 1.021-1.188, p = 0.013; OR: 1.134, 95%CI: 1.024-1.255, p = 0.015). IL-13 also displayed a significant positive correlation with UC (OR: 1.099, 95%CI: 1.018-1.186, p = 0.016). Stem cell factor (SCF) was suggested to be associated with the development of both CD and UC (OR: 1.032, 95%CI: 0.973-1.058, p = 0.012; OR: 1.038, 95%CI: 1.005-1.072, p = 0.024).

**Conclusion:**

This study proposes that IL-13 may be a factor correlated with the etiology of IBD (CD and UC), while MIF just be specifically associated with CD. Additionally, SCF appears more likely to be involved in the downstream development of IBD (CD and UC).

## 1 Introduction

Inflammatory bowel disease (IBD) is a nonspecific immune-mediated, chronic recurrent gastrointestinal disease and can be subcategorized into Crohn’s disease (CD), ulcerative colitis (UC), and idiopathic colitis (1). The global incidence of UC is on the rise, and it is projected that by 2030, the prevalence rate among Western populations will reach 1%. This poses a significant burden on both global health and the economy (2, 3). Until now, a comprehensive understanding of the etiology and pathogenesis of UC has eluded researchers. The factors implicated include genetic susceptibility, compromised gut mucosal barriers, environmental influences such as increased hygiene standards, urban living, dietary elements, and the dysregulation between gut microbiota and mucosal immunity, any of which may contribute to the onset of UC (4). The primary focus of numerous studies has been on investigating the involvement of immune responses in the pathogenesis of IBD (5, 6). With prolonged activation of the immune system within the intestinal mucosa, it promotes the release of various biomarkers, such as cytokines, including interleukin, chemokine and tumor necrosis factor (7). In canine IBD, there is an initiation of a pro-inflammatory pathway leading to Th cell differentiation, primarily driven by microbial dysbiosis. This imbalance in the microbial community stimulates the generation of mainly pro-inflammatory factors, notably IL-1b. Furthermore, mutations in pattern recognition receptors (PRRs), for example Toll-like receptor 5 (TLR5), heighten the response to flagellin. With dysbiosis characterized by increased Enterobacteriaceae, flagellin expression intensifies, enhancing the mucosa’s pro-inflammatory reactions. Consequently, the inflammatory cytokines induce structural changes in epithelial cells, notably increasing permeability due to augmented leakage through tight junctions. This increased permeability establishes a vicious cycle, allowing more bacteria to breach the mucosal barrier, perpetuating the self-reinforcing cycle of inflammation (8). In CD, there’s a higher expression of Th1 cell-related cytokines, particularly IFN-γ and IL-2, compared to both UC and individuals without the condition. Conversely, mucosal cells in UC exhibit a tendency to produce Th2-type cytokines like IL-5 and IL-13 (9–12). Multiple studies have indicated an elevated synthesis of Th17 cell-related cytokines, such as IL-17a and IL-17F, by mucosal T cells in both CD and UC (13). Meanwhile, a systematic review showed that six chemokines, including CCL2 (MCP-1), CCL11 (EOTAXIN), CCL26 (EOTAXIN-3), CXCL1 (GROa), CXCL8 (IL-8) and CXCL10 (IP-10), as biomarkers of CD activity are controversial (14). Though some observational studies have endeavored to clarify the connections between inflammatory cytokines and IBD. The conclusions derived from these investigations might be influenced by unforeseen confounding factors or reverse causation, complicating the establishment of definite causal correlations.

Mendelian randomization (MR) is recognized as the analytical method that infers the causal impact of an exposure on an outcome by utilizing genetic variations in non-experimental data (15). As alleles are randomly assigned during meiosis, Mendelian randomization (MR) has the capacity to minimize traditional confounding variables and reverse causation, thereby offering improved evidence for causal inference (16). Conducting a two-sample MR analysis permits researchers to assess the associations between the instrumental variables and both exposure and outcome across two distinct population samples, thereby improving the test’s applicability and effectiveness (17). In this study, we first extracted valid genetic variants from the published genome-wide association study (GWAS) summary data of 18 inflammatory cytokines in order to investigate their associations with IBD, and then the direction of causation was further explored by reversing the exposures and outcomes.

## 2 Methods

### 2.1 Study design

**Figure 1:**
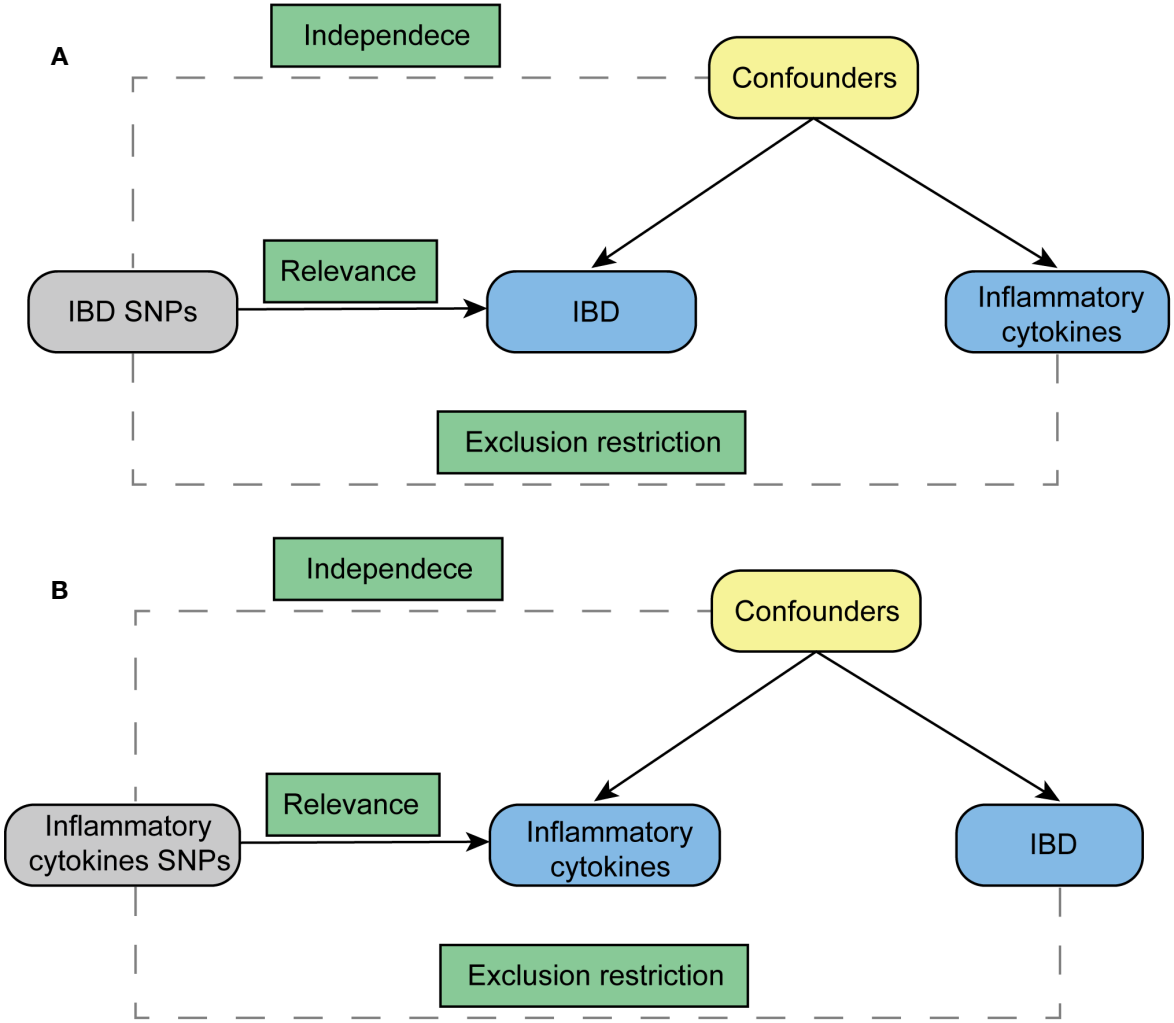
Diagram for key assumptions of MR analyses. **(A)** IBD SNPs were used as the genetic instruments to investigate the causal effect of IBD on inflammatory cytokines. **(B)** Genetic instruments in the form of inflammatory cytokine SNPs were utilized to explore the causal relationship between inflammatory cytokines and IBD. The lines with arrows signify the association of genetic instruments (SNPs) with the exposure, affecting the outcome solely through the exposure. Meanwhile, dashed lines represent the independence of the genetic instruments (SNPs) from any confounding variables concerning the outcomes. IBD stands for inflammatory bowel disease.

The foundation of this study relies on a genetic association database derived from GWAS summary datasets (https://gwas.mrcieu.ac.uk/). Multiple single-nucleotide polymorphisms (SNPs) were chosen to represent genetic variability and used as instrumental variables in a two-sample MR analysis. Three primary hypotheses were established as outlined below (**Figure 1**): 1. The instrumental variables have a direct association with the exposure; 2. The instrumental variables are not influenced by any confounding variables; 3. Genetic variants solely impact outcomes through their effect on exposure (18). MR analysis was employed to evaluate the bidirectional causal connections between inflammatory cytokines and IBD, encompassing both UC and CD.

### 2.2 Data sources

Both datasets utilized in this MR analysis were sourced from publicly available summarized GWAS data. GWAS data of IBD, containing CD and UC, came from a meta-analysis study. 12194 CD cases (28072 healthy controls), and 12336 UC cases (33609 healthy controls) were available in this data set, with corresponding GWAS IDs of ebi-a-GCST004132 and ebi-a-GCST004133, respectively. For inflammatory cytokines, the data was from the study providing genome variant associations with cytokines and growth factors in 8,293 Finnish individuals. This study combined the results from The Cardiovascular Risk in Young Finns Study (YFS) and FINRISK surveys. The average participant ages are 37 years for YFS study and 60 years for FINRISK survey. There would be no overlap in population selection between the exposure group and the outcome group.

### 2.3 Instrumental variable selection

Initially, we established the genome-wide significance threshold as p < 5 × 10^-8^ to pinpoint highly associated SNPs linked with IBD and inflammatory cytokines. However, due to the limited number of identified SNPs for certain inflammatory cytokines when they were considered as the exposure, a higher cutoff (p < 5 × 10^-6^) was adopted. Next, for the purpose of evading linkage disequilibrium, we conducted SNP clumping (kb = 10,000, r^2^ = 0.001). Palindromic SNPs were omitted due to uncertainty regarding their alignment in the same orientation for both exposure and outcome in the GWAS of systemic inflammatory regulators. Finally, we assessed the potency of each SNP utilizing the F-statistic, which integrates the extent and accuracy of the genetic impact on the trait: F = R^2^(N - 2)/(1 - R^2^), where R^2^ signifies the proportion of the trait’s variance elucidated by the SNP, and N denotes the sample size of the GWAS encompassing SNPs associated with the trait (19). The R^2^ values were estimated using the formula R^2^ = 2×EAF×(1 - EAF)×β^2^. The effect allele frequency (EAF) of the SNP is denoted as EAF, and β represents the estimated effect of the SNP on the trait. SNPs with an F-statistic less than 10 were excluded, as an F-statistic greater than 10 indicated ample strength, ensuring the credibility of the SNPs.

### 2.4 Statistical analysis

Main MR analysis was conducted using the inverse variance weighted (IVW) method. In the MR analysis, multiplicative random effects were applied when utilizing more than three SNPs or in cases of heterogeneity. Other MR methodologies employed to verify result consistency encompassed the weighted median, MR-Egger, simple mode, and weighted mode. Heterogeneity among SNPs was evaluated using the Cochran Q test analysis of IVW and MR-Egger. The MR-Egger intercept test served to identify potential horizontal pleiotropy (version 4.2.2).

## 3 Results

### 3.1 Influence of 18 inflammatory cytokines on IBD

**Figure 2:**
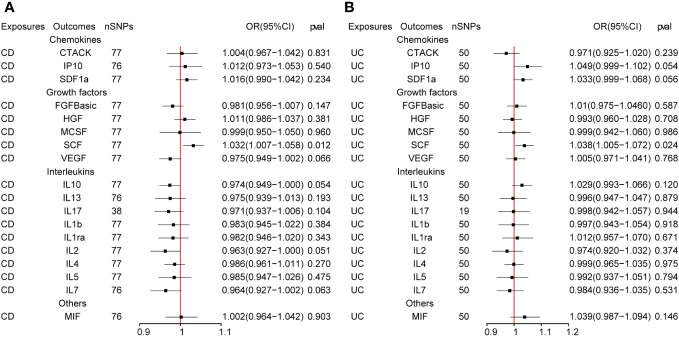
**(A, B)** The presented figures represent Mendelian randomization estimates illustrating the causal impacts of CD and UC on inflammatory cytokines. The estimates are displayed as OR and 95% CIs derived from bidirectional Mendelian randomization analyses. OR, odds ratio. 95% CI, 95% confidence interval.

**Figure 3:**
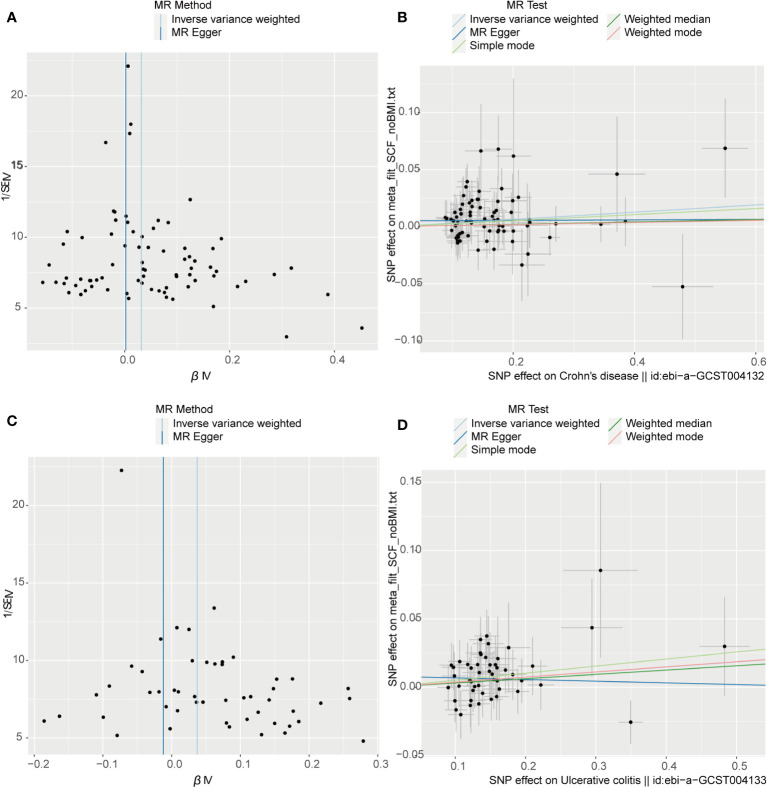
Visual aids like scatter plots and funnel plots were employed to illustrate the causal effects of CD and UC on inflammatory cytokines. **(A, C)** The funnel plots depict the inverse variance weighted MR estimates of single-nucleotide polymorphisms associated with CD and UC against cytokines versus 1/standard error (1/SEIV). **(B, D)** Black dots display individual inverse variance (IV) associations with the risk of CD and UC versus individual IV associations with cytokines. The 95%CI of odd ratio for each IV is shown by vertical and horizontal lines. The slope of the lines represents the estimated causal effect of the MR methods.

The outcome of the MR analysis indicated a significant positive correlation between genetically predicted IL-13 (OR: 1.101; 95%CI: 1.021-1.188; p = 0.013) and macrophage migration inhibitory factor (MIF) (OR: 1.134; 95%CI: 1.024-1.255; p = 0.015) with CD (**Figure 2A**). IL13 (OR: 1.099; 95%CI: 1.018-1.186; p = 0.016) exhibited a notable positive correlation with UC (**Figure 2B**). **Figure 3** displays the scatter plots and funnel plots depicting the Mendelian randomization analyses for IL13 and MIF in IBD. The results from sensitivity analysis indicated that the MR-Egger regression analysis indicated no presence of horizontal pleiotropy, while the Cochran Q test demonstrated the absence of heterogeneity among IVs (**Supplementary Table S1**). Details of the SNPs are also presented in **Supplementary Table S1**. While the forest plots and leave-one-out sensitivity analyses of all suggestively significant regulators are presented in **Supplementary Figure S1**.

### 3.2 Influence of IBD on 18 inflammatory cytokines

**Figure 4:**
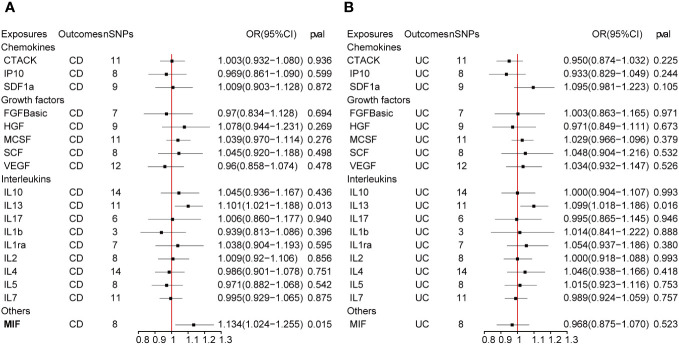
**(A, B)** represent mendelian randomized estimates of the causal effect of the ILs and chemokines on CD and UC. Estimates are presented as odds ratios (ORs) and 95% CIs from bidirectional mendelian randomization analyses.

**Figure 5:**
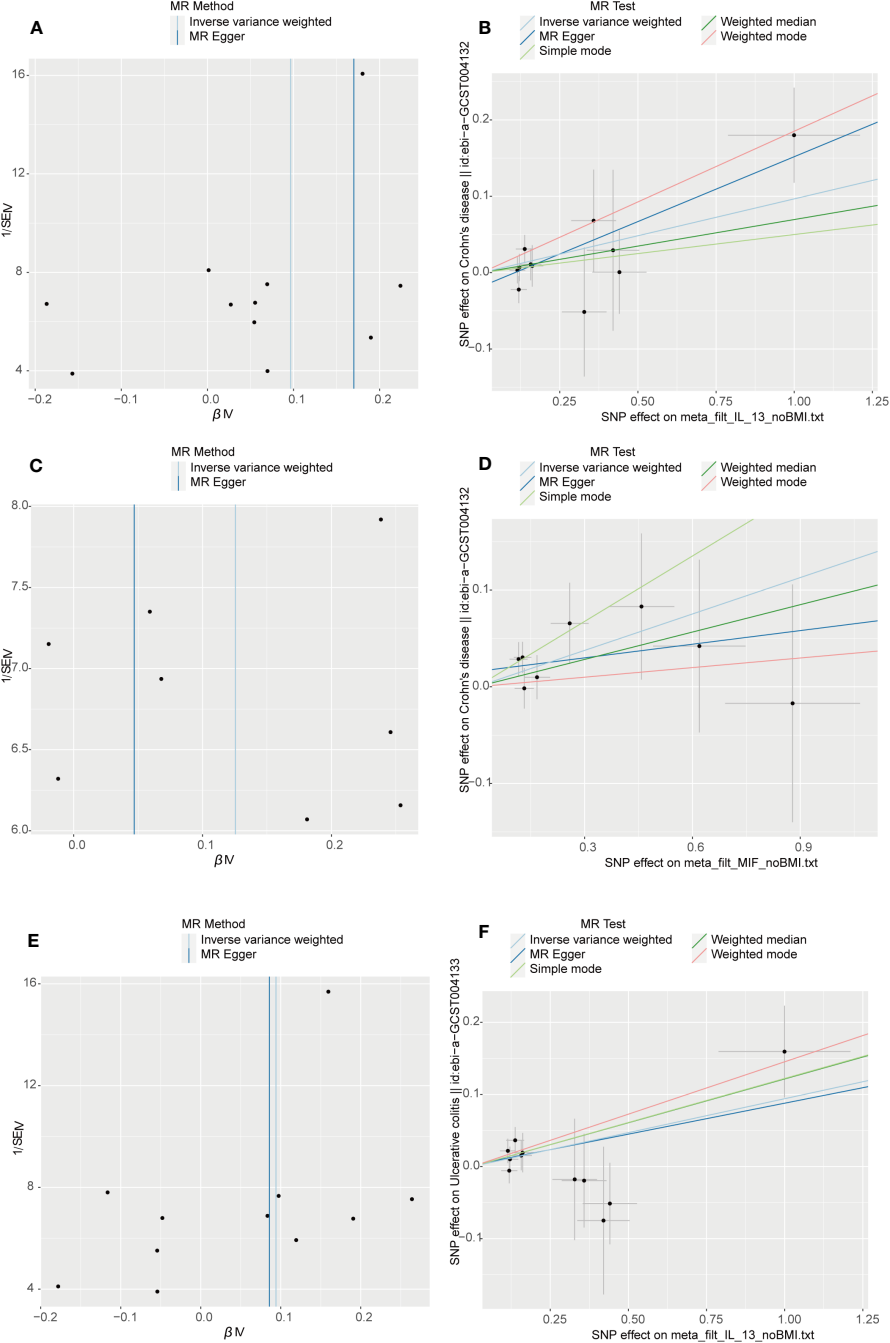
Visual aids like scatter plots and funnel plots were employed to illustrate the causal effects of inflammatory cytokines on CD and UC. **(A, C, E)** The funnel plots illustrate the inverse variance weighted MR estimates of each cytokine single-nucleotide polymorphism with CD and UC against 1/standard error (1/SEIV). **(B, D, F)** Black dots display individual inverse variance (IV) associations with the risk of cytokines versus individual IV associations with CD and UC. Vertical and horizontal lines depict the 95% confidence intervals (CI) of the odds ratio for each IV. The slope of these lines indicates the estimated causal effect determined by the MR methods.

**Figure 4** presents the outcomes from the reverse MR analysis regarding the causality between IBD and inflammatory cytokines. The results obtained from the IVW method suggested a correlation between an increased level of Stem cell factor (SCF) and CD (OR: 1.032; 95% CI: 1.007-1.058; p = 0.012). Meanwhile, UC was also suggestively correlated with an elevated level of SCF (OR: 1.060; 95% CI: 1.006-1.118; p = 0.028). The scatter plots and funnel plots of SCF are displayed in **Figure 5**. The results from sensitivity analysis indicated that the MR-Egger regression analysis indicated no presence of horizontal pleiotropy, while the Cochran Q test demonstrated the absence of heterogeneity among IVs (**Supplementary Table S1**). **Supplementary Figure S2** includes forest plots and leave-one-out sensitivity analyses for all regulators that showed suggestive significance.

## 4 Discussion

A recent Mendelian randomized study delved into the causal connections between five interleukins, six chemokines, and IBD. The findings indicated significant positive correlations of IL-16, IL-18, and CXCL10 with IBD, contrasting with IL-12p70 and CCL23, which showed significant negative correlations. Additionally, IL-16 and IL-18 suggested an increased risk of UC, while CXCL10 hinted at an increased risk of CD (20).

We expanded the number of inflammatory cytokines (which includes ILs, chemokines, growth factors and others) and explored the causal relationship between more inflammatory factors and IBD. To understand the causal relationship between IBD and inflammatory cytokines, we used publicly aggregated GWAS data for two-way MR analysis. In the forward MR analysis, elevated levels of IL-13 and MIF were associated with increased risk of CD, whereas IL-13 was also linked to an increased risk of UC. In our reverse MR analysis, CD and UC were suggestively associated with elevated levels of SCF.

Differentiating between CD and UC predominantly depends on the localization of inflammatory lesions and the specific cytokine involvement in their pathogenesis. CD manifests as a segmental, transmural disorder that can impact any segment of the gastrointestinal tract, while UC is identified by superficial, continuous mucosal ulcers restricted to the colon. Dysregulation between pro- and anti-inflammatory cytokines is widely acknowledged in both CD and UC (21). In CD, an association exists with a T helper type 1 (Th1) and T helper type 17 (Th17) immune response (22), leading to the secretion of diverse pro-inflammatory cytokines, such as IFNγ/IL12 and IL23/IL17, which encompass IL18, IL2, IL1, IL21, and IL22. Conversely, UC demonstrates a distinct Th17 and an altered Th2 cytokine profile, characterized by IL13 and IL5. Moreover, both Th1 and Th2 cells, alongside macrophages in both types of IBD, contribute to the production of IL6 and tumor necrosis alpha (TNFα) (23). Genetic polymorphisms in cytokine and cytokine receptor genes may significantly impact the progression of the inflammatory cascade, potentially elevating the susceptibility to developing IBD.

IL-13 is a typical Th2 cytokine produced from CD-1-reactive NKT cells, and its secretion mediates epithelial barrier dysfunction. An increase of IL-13 in lymphocytes of the lamina propria in the affected area of UC represented a significant role of the Th2 immune response in UC pathogenesis. Furthermore, IL-13 was responsible for impairment of mucosal permeability that resulted in epithelial barrier damage, and There were alterations observed in the tight junctions of intestinal epithelial cells. In patients with UC, IL-13 showed a significant increase within apoptotic cells and the corresponding apoptotic area (The Th2 colitis model, Oxazolone colitis, resembling ulcerative colitis, is mediated through IL-13-producing NK-T cells). In the lamina propria’s mononuclear cell culture, IL-13 heightened ion flux, leading to alterations in cellular tight junctions. IL-13 exerted an influence on mucosal repair, artificially reducing the rate of mucosal repair by 30% upon its addition to the mucosal lesions (24). Two papers published in 2004 and 2005 reported that ex vivo stimulated lamina propria T cells, obtained from resected specimens of UC patients, exhibited heightened protein levels of IL-13 compared to individuals with CD and those who were healthy (24, 25). The inflammatory infiltrate of TNF and IL-13 triggers epithelial-to-mesenchymal transition and upregulation of matrix metalloproteinases, resulting in tissue remodeling and the formation of fistulas (26–28). In a Polish population, the presence of IL13 -1112 CT (rs1800925) genotypes indicated an increased likelihood of both IBD and UC occurrence (29).

Initially identified as a factor released by T cells, macrophage migration inhibitory factor (MIF) inhibits the random migration of macrophages (30). Later investigations disclosed that MIF acts as a pro-inflammatory factor., which has important roles in various chronic inflammatory diseases and immune disorders, including UC (31). Some studies showed the capacity of MIF to induce increased functional capacity of DC, and to produce IL-1b and IL-8 from monocytes and DC, indicate a role of MIF in the induction and/or perpetuation of the inflammatory environment in UC and CD (32).

Stem cell factor, also recognized as SCF, KIT-ligand, or steel factor, is a pleiotropic cytokine that governs regulatory impacts on inflammation, tissue remodeling, and fibrosis by binding to its receptor c-KIT (33, 34). SCF is extensively recognized for its role in governing the survival, proliferation, migration, and differentiation of hematopoietic progenitors, melanocytes, and germ cells. Recent investigations have indicated the expression of SCF in dermal and intestinal epithelial cells (35–37). Reports have indicated elevated SCF expression in the inflamed mucosa of individuals with IBD, including SCF248 (a 248 amino acid cleavable form) (38, 39).

Within the cascade of inflammatory events leading to the development of IBD, the involvement of inflammatory cytokines is intricate and potentially interactive. However, MR analysis can isolate their individual impacts and assess the relationship between IBD and these cytokines solely from a genetic standpoint. Nevertheless, our study faces limitations. Primarily, our findings stem from statistical analysis; further validation through extensive basic and clinical research is imperative. Additionally, while restricting the study to individuals of European descent diminishes population structure bias, it could constrain the generalizability of our findings to other populations.

## 5 Conclusion

The publicly available data information from the GWAS database was sourced and analyzed in this study to evaluate the causal relationship between IBD and ILs, and IBD and chemokines, by bidirectional MR analysis. Our results have shown that levels of IL-13 increase the risk of CD and UC, while MIF increase the risk of CD. CD and UC were suggestively correlated with an elevated level of SCF. The underlying mechanism behind these outcomes remains unclear, necessitating further investigation to substantiate our findings.

## Data availability statement

Publicly available datasets were analyzed in this study. This data can be found here: https://doi.org/10.5523/bris.3g3i5smgghp0s2uvm1doflkx9x.

## Author contributions

ZS: Writing – review & editing. XL: Writing – review & editing. JX: Writing – original draft. FH: Writing – original draft. NW: Writing – original draft. YH: Writing – review & editing. JY: Writing – review & editing.
