# Supplementary figures and images for: Associations of inflammatory cytokines with inflammatory bowel disease: a Mendelian randomization study
Source: Front Immunol. 2024 Jan 15;14:1327879. doi: 10.3389/fimmu.2023.1327879 (PMC10822978; doi:10.3389/fimmu.2023.1327879)

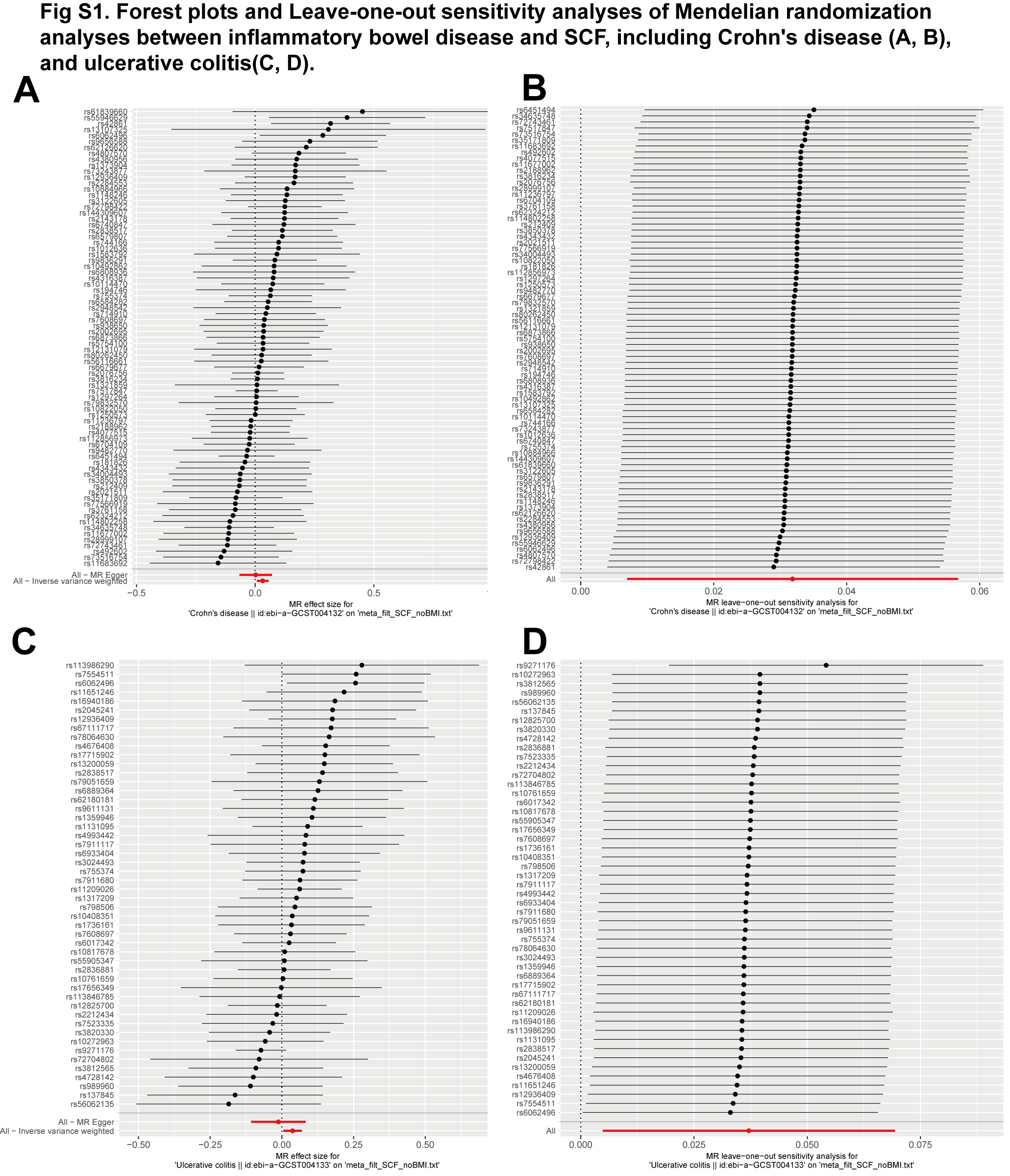

Supplement: Supplementary file 1 [file Image_1.tif]

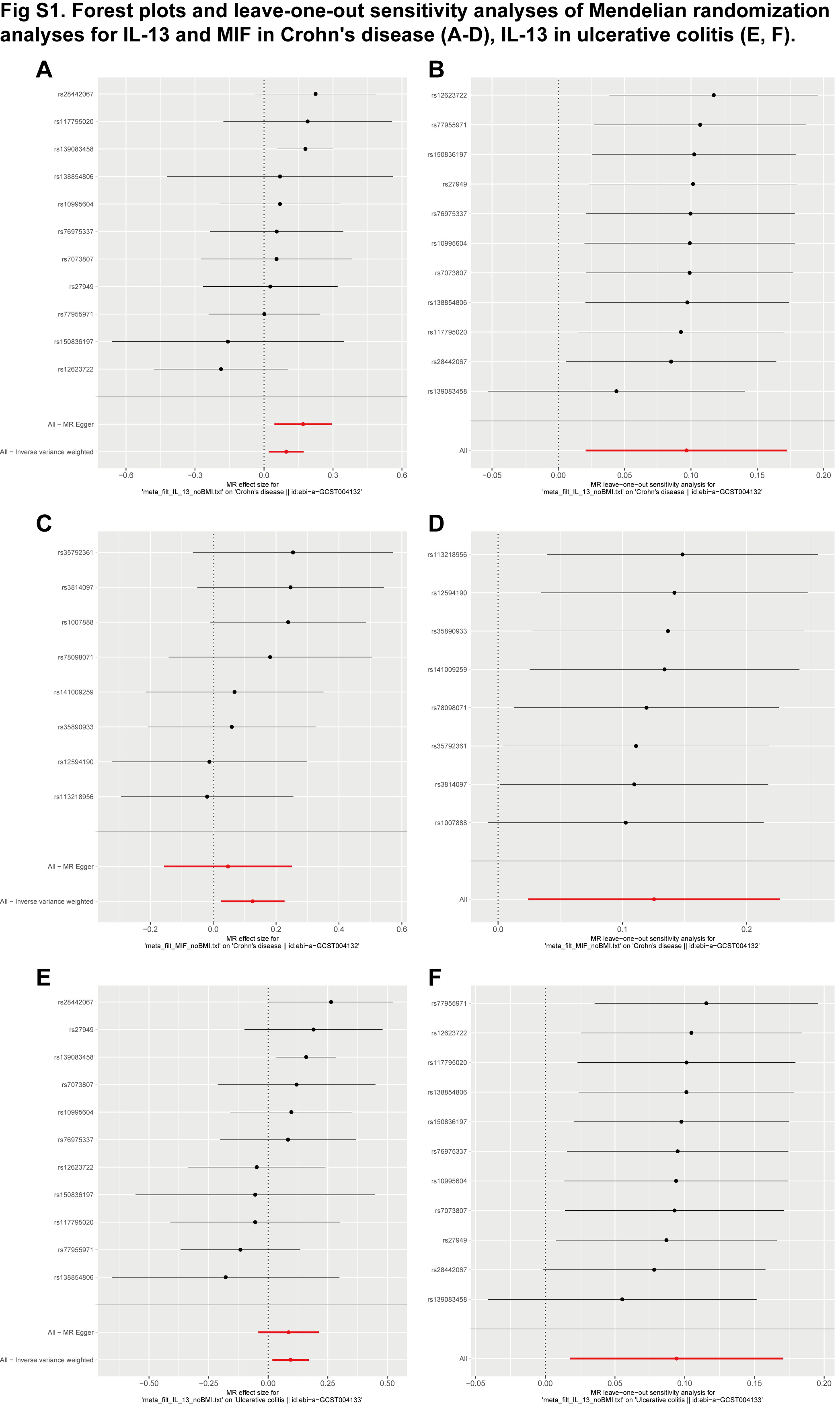

Supplement: Supplementary file 2 [file Image_2.tif]
